# On the Tussis Convulsiva, and a More Efficacious Treatment of This Disease

**Published:** 1806-03

**Authors:** 


### On the Tussis Convulsiva, and a more efficacious Treatment of this Disease; by Dr. Jacobi.

THE Tussis Convulsiva has often been reckoned among the most obstinate diseases, baffling all medical skill, and only giving way to time, or a favourable change in the constitution. How far this objection may be justly founded, we shall not discuss, but gratefully acknowledge every endeavour to establish a more effectual cure of this tiresome complaint. According to the doctrines of the ancient school, the tussis convulsiva was treated partly as a sthenic, and partly as an asthenic disease. It was almost universally believed to be impossible to cure the tussis without emetics, which, of course, were often repeated. The consequence was, that in some instances after the emetic emetic had operated, the cough became milder, or ceased for a time, but soon after it returned with greater violence. After this medicine, stimulants or antispasmodics, often very untimely, were administered.

Dr. J. questions whether the tussis convulsiva originates from a specific contagion? But even supposing this to be granted, why may not diseases spreading through contagion, be shortened, in the most efficacious manner? Why is medicine to give way to them? He is of opinion that this disease is no more than any other morbus convulsivus, deriving only its peculiar form from the organs affected. In general, the tussis convulsiva is of the asthenic kind, and very seldom of a sthenic diathesis; which being obvious, must be treated accordingly. The asthenic cough affects mostly the stomach, the diaphragm and the lungs, with returning convulsive paroxysms of these organs; in consequence of which more phlegm is secreted, and generally thrown out by vomiting, at the end of every violent paroxysm. As the illness proceeds, the secretion of phlegm and the vomiting increase. Towards evening a fever associates; and delicate children often have a bleeding from the nose or the fauces.

The Doctor begins the cure with bitter roborant medicines, combined with the most powerful stimulants; musk, camphor, tincture of cinnamon, æther of vitriol, &c

To give a more distinct idea of his practice, we shall transcribe two of his cases.

Case I.—A girl, eight years old, of a weak constitution, pale faced, the abdomen much distended, was attacked with a very severe tussis convulsiva, often threatening suffocation. She became sick as soon as she eat any thing, and bled frequently from the nose and fauces. A nourishing, light, and suitable diet was ordered, and the following medicine:

℞. Extr. lign. quass. drachm. ij. solv. in aq. menthæ piper. unc. v. aquæ cinam. s. unc. j. syr. commun. unc. jß. liquor anodyn. Hofm. drachm ij. m. s. A table spoon full to be taken five or six times; and at the same time, ℞. Moschi opt. gr. vj. sacchar. alb. drachm. ij. divid. in vj. partes æquales; to be taken with the former medicine.

She continued the medicine, and the quantity of musk was increased the next day to three grains in every powder. Within five days the violence of the cough was much abated, the paroxysms returned less frequent, and the bleedings and vomitings almost ceased. From economical views, after the fifth day the camphor was substituted, and from this time she took mosch. gr. xvj. camph. gr. xiij. in four portions. At the expiration of ten days the tussis convulsiva was transformed into a common catarrhal cough, with slight expectoration. She now took ℞. Camphor. pulv. gr. xxij. sacchar. alb. gr. v. divid. in iv. partes æquales, the other medicines being put aside, and in the third week the cough entirely left her.

Case II.—A boy, four years and a half old, very thin and poorly, suffered by the tussis convulsiva for several weeks in a high degree. Without any previous emetic, Dr. J. prescribed, ℞. Extract. cort. Peruv. dr. ij. sacchar. unc. j. ol. menth. pip. dist. gtt. viij. aquæ deft. unc. jß. tinct. cinam. unc. j. æther. vitriol. ℈j. of which he was to take a spoonful every two hours, and every three hours the following powder: ℞. Mosch. gr. iv. sacchar. ℈iv. M. div. in iv. partes æquales. The quantity of musk was augmented every day to two grains and a half, and the medicine continued. The result was such, that after the expiration of the tenth day the disease was almost entirely exterminated; the musk was now omitted, but the first medicine, with a little alteration, for some time continued.

